# Impact of Double Reflex Testing and Linkage to Treatment on Clinical Outcomes of Chronic Hepatitis Delta Virus Infection in the United States

**DOI:** 10.1111/jvh.70119

**Published:** 2025-12-17

**Authors:** Robert J. Wong, Robert G. Gish, Ira M. Jacobson, Joseph K. Lim, Marvin Rock, Csilla Kinyik‐Merena, Hanxiao Ma, Nathaniel Smith, Chong Kim

**Affiliations:** ^1^ Stanford University School of Medicine Stanford California USA; ^2^ Hepatitis B Foundation Doylestown Pennsylvania USA; ^3^ New York University Grossman School of Medicine New York New York USA; ^4^ Yale School of Medicine New Haven Connecticut USA; ^5^ HEOR—Global Value and Access, Gilead Sciences, Inc. Foster City California USA; ^6^ Maple Health Group New York New York USA

**Keywords:** cirrhosis, diagnosis, liver complications, liver‐related mortality, screening

## Abstract

Hepatitis D virus (HDV) screening rates in the United States are low. We evaluated the impact of double reflex‐based HDV testing on HDV‐related morbidity and mortality in the United States. A Screen and Treat model simulated the HDV screening cascade (decision tree) and assessed the natural history of HDV (Markov model) over a 5‐year time horizon from a United States third‐party payer perspective, for patients positive for HBV. The number of patients diagnosed with HDV and liver‐related outcomes were compared under double reflex screening (100% patients screened for HDV antibodies and HDV RNA) and current practice (11% patients screened for HDV antibodies and 25% anti‐HDV‐positive patients for HDV RNA). Implementation of HDV double reflex testing predicted a 3655% increase in patients diagnosed versus current practice (*n* = 7231 vs. *n* = 193, respectively). The number of predicted occurrences of all liver‐related outcomes over 5 years is lower with double reflex testing versus current practice (difference in numbers of events: −7% for compensated cirrhosis, −40% for decompensated cirrhosis, −23% for hepatocellular carcinoma, −34% for liver transplantation, and −32% for liver‐related deaths). Results of scenario analyses with varying HBV prevalence, treatment received, or treatment rates were similar. Simulation of double reflex testing predicted earlier detection of HDV patients, increased numbers of patients diagnosed and treated, and reduced rates of disease progression, liver‐related complications and deaths. These findings highlight the need for implementing strategies to improve HDV screening and linkage to care and treatment in the United States.

**Trial Registration:** ClinicalTrials.gov identifier: NCT03852719

## 1 Introduction

Hepatitis D virus (HDV) is a defective RNA virus which only infects people positive for the hepatitis B surface antigen (HBsAg) [1]. HDV is considered the most severe form of viral hepatitis [1, 2]. Compared to mono‐infection with the hepatitis B virus (HBV), HDV co‐infection carries a greater risk of morbidity and mortality and is associated with accelerated progression to liver cirrhosis, decompensation and hepatocellular carcinoma (HCC) [1, 2, 3, 4, 5]. In the United States, the estimated prevalence of HDV co‐infection among the HBV population is 4.6% [6], with a recent meta‐analysis estimating that over 75,000 individuals were living with HDV in the United States in 2022 [7]. However, HDV prevalence is believed to be underestimated [6, 8], which is likely a result of insufficient testing among individuals with HBV [5, 6, 8, 9], and heterogeneity in the performance of serological tests and sampling [2, 10].

Rates of HDV screening in the United States are presently low, estimated at 6%–20% of patients with chronic HBV [11, 12, 13, 14]. Suboptimal diagnosis of HDV consequently complicates disease management as patients typically present with advanced disease and have limited treatment options [5]. Increased HDV screening is needed to improve HDV diagnosis, the benefits of which include reducing the risk of HDV transmission, disease progression and development of complications, and reducing the subsequent need for HDV treatment [5, 15, 16].

Double reflex testing for HDV is a screening approach which includes reflex (automatic) testing for HDV total antibodies for all patients positive for HBsAg, followed by reflex HDV RNA testing for all anti‐HDV‐positive patients [15, 17]. Reflex testing for HDV antibodies led to a 3.9 to 5‐fold increase in HDV diagnoses in academic hospitals and primary care centers in Spain and Italy, compared with usual care [18, 19]. A recent meta‐analysis has also shown that HDV RNA positivity was associated with increased risks of advanced liver disease among patients who tested positive for HBsAg and HDV antibodies [3]. In particular, compared to patients who were negative for HDV RNA, those positive for HDV RNA had significantly higher risks of progressing to decompensated cirrhosis (DCC) (hazard ratio [HR] 3.82, 95% confidence interval [CI] 1.60–9.10), HCC (HR 2.97, 95% CI 1.87–4.70), liver transplantation (LT) (HR 7.07, 95% CI 1.61–30.99), and liver‐related mortality (HR 3.78, 95% CI 2.18–6.56) [3].

The American Association for the Study of Liver Diseases (AASLD) currently recommends HDV antibody screening only in HBsAg‐positive patients who are at risk; these include people who inject drugs, men who have sex with men, people at risk of acquiring sexually transmitted diseases, and people with hepatitis C virus (HCV) or human immunodeficiency virus infection [20]. By contrast, other clinical guidelines recommend that all HBsAg‐positive individuals should be screened for HDV total antibodies, and that HDV RNA should be tested in all who were positive for HDV antibodies [5, 21]; other causes of chronic liver disease should also be systematically examined in patients with HBV [22].

We previously developed a natural history model for HDV and evaluated the impact of bulevirtide treatment versus best supportive care, and results showed that patients who received bulevirtide experienced improved liver disease outcomes, including reduced disease progression and liver‐related mortality [23]. In light of the low HDV screening rates in the United States and the anticipated benefits of improved HDV diagnoses, we adapted the previous model and assessed the impact of double reflex testing for HDV compared with current practice on HDV‐related morbidity and mortality in the US, using a Screen and Treat simulation modelling approach.

## 2 Methods

### 2.1 Model Overview

**FIGURE 1:**
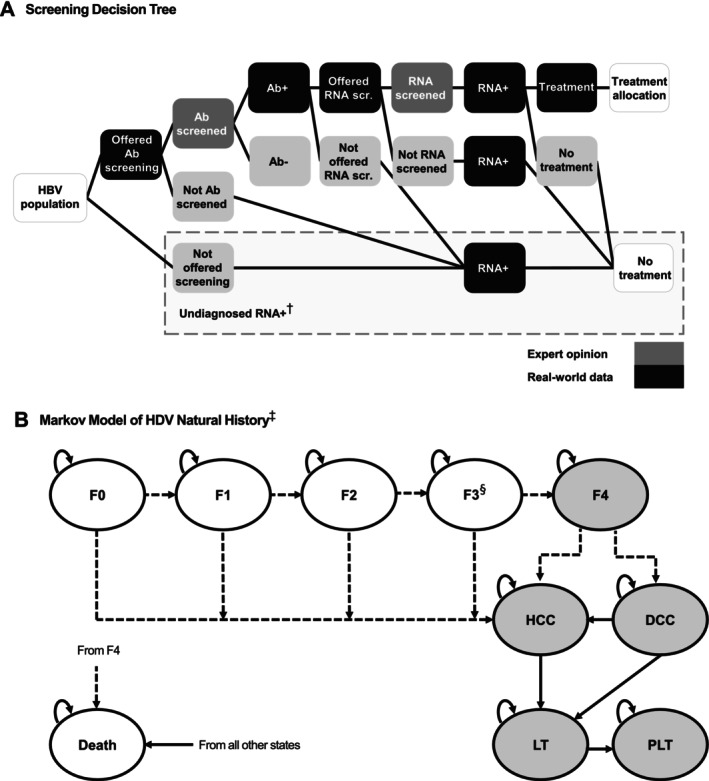
Model schematic. (A) Screening decision tree, (B) Markov model of HDV natural history. ^†^Patients could be RNA+ even if they were not screened; only patients identified by RNA screening could receive treatment. ^‡^Dashed arrows: Rate of disease progression impacted by achieving response; solid arrows: Transition probability not impacted by achieving response; grey health states: Higher mortality rates than the general population. The Markov model of HDV natural history was described in Kaushik et al. [23]. ^§^Patients achieving response could also regress from F4 → F3 and F3 → F2. Ab, antibody; DCC, decompensated cirrhosis; F, fibrosis stage; HBV, hepatitis B virus; HCC, hepatocellular carcinoma; LT, liver transplantation; PLT, post‐liver transplant; scr., screened.

The Screen and Treat model follows patients with HDV through the lifetime of their disease and consists of a screening decision tree component, which simulates the HDV screening cascade, followed by a Markov model to assess the natural history for HDV patients (Figure 1). The model estimated the incidence of liver complications over a 5‐year time horizon from the United States third‐party payer perspective, with outcomes discounted at 3%. The patient population at model entry consists of adults positive for HBsAg (67% male and mean age of 40.2 years at baseline) [23].

The screening decision tree accounts for patients who were screened for HDV and the impact of treatment in those who tested positive for HDV RNA and received treatment. In the decision tree, patients were screened for HDV and could be either diagnosed or remain undiagnosed under two screening approaches: current practice and double reflex testing. Under current practice, 10.7% of HBsAg‐positive patients were offered screening with the total HDV antibody test, and 25.0% of anti‐HDV‐positive patients were offered HDV RNA molecular testing. Based on unpublished internal data, an estimated 1.14% of HDV RNA‐positive patients were predicted to initiate treatment. Under double reflex testing, all HBsAg‐positive patients were offered screening with the total HDV antibody test, and all anti‐HDV‐positive patients were screened for HDV RNA. It was assumed that 85% of HDV RNA‐positive patients would initiate treatment. These approaches to HDV screening were validated by expert opinion.

The Markov cohort model simulates the burden of HDV disease and was based on a previously published model which included Markov state transition models typical of other viral hepatitis models [23]. In the Markov model, over the course of a simulation, patients could achieve spontaneous (i.e., HBsAg seroconversion) or treatment‐induced responses (i.e., HDV RNA and alanine aminotransferase [ALT] level reduction) or develop long‐term liver complications (compensated cirrhosis [CC], DCC, HCC, LT), or death. The model links the efficacy of treatments using a composite endpoint to the slowing of disease progression; efficacy was based on HDV‐RNA undetectability or 2log decline and ALT normalisation, which was a composite endpoint in the Phase 3 MYR 301 study (NCT03852719) [24]. Slowed disease progression within the model was expected to reduce HDV‐related morbidity and mortality; this is in line with a prior economic model for chronic HDV [23], where patients who responded to treatment had slower rates of progression based on hazard ratios sourced from a recent meta‐analysis of natural history data between HDV RNA negative versus HDV RNA positive patients in regards to progression to advanced liver disease [3]. The hazard ratios were applied to baseline natural history progression rates used for non‐responders to determine reduced rates of progression for treatment responders. Patients entered the model at a set baseline distribution of fibrosis stages, based on published data [6, 25] and assumptions.

Patients were assigned treatment to pegylated interferon‐alpha (PEG‐IFN‐α) for current practice or bulevirtide (HEPCLUDEX) for double reflex testing in the base case. At Weeks 24 and 48 of treatment, a percentage of patients under each treatment reached the composite endpoint based on data from MYR 301 [24]. Patients who achieved the composite endpoint were considered responders and those who did not achieve the composite endpoint were considered non‐responders.

### 2.2 Model Inputs

Inputs for screening parameters in the decision tree are shown in Table 1. The proportion of HBsAg‐positive patients was estimated at 0.25%, based on the published United States prevalence of chronic HBV infection (0.72% of the total population) [27] and the proportion of HBV patients who are diagnosed (35%) [28]. Proportions of HBsAg‐positive patients receiving HDV antibody and RNA testing, rates of RNA positivity, and proportions of patients receiving treatment were based on published data [13], real‐world data [14], expert opinion, and assumptions. Published data were reviewed to inform assumptions regarding current testing rates and epidemiological inputs (e.g., in regards to the proportion of patients with chronic HDV within those with chronic HBV and the distribution of cirrhosis in diagnosed and undiagnosed patients). Given that there are limited available data, suggested model inputs based on identified data were validated with one‐on‐one discussions with clinical experts familiar with the treatment and management of patients with chronic HDV. In these discussions, suggested data were reviewed with experts; where assumptions and evidence gaps remained after discussion, suggestions for scenario analyses within the model were provided and prioritised for inclusion within the study. Inputs regarding model efficacy assumptions, natural history, and other non‐testing related inputs are as described in a previously published model [23].

**TABLE 1:** Inputs for screening parameters (decision‐tree).

Model input	Current practice			Double reflex testing		
	Value (%)	*N* (range)	References	Value (%)	*N* (range)	References
US population^a^	/	335,531,299	US Census Bureau [26]	/	335,531,299	Per current practice
% HBsAg+	0.25	845,539	Wong et al. [27], CDA Foundation [28]	0.25	845,539	Per current practice
Ab screening rate	10.65	90,079	John et al. [13]	100	845,539	Assumption
% Accepting Ab screening	88.78	79,970	Wong et al. [14]	88.78	750,653	Per current practice
Ab positivity rate	1.91	1530	Wong et al. [14]	1.91	14,361	Per current practice
% Ab+ receiving RNA screening	25.00	382	Wong et al. [14]	100	14,361	Assumption
% Accepting RNA screening among Ab+	90.00	344	Expert opinion	90.00	12,925	Per current practice
RNA positivity rate	55.95	193	Wong et al. [14]	55.95	7231	Per current practice
% Receiving treatment^b^	1.14	2	Real‐world database; data on file [29]	85.00	6146	Assumption

Abbreviation: Ab, antibody.

^a^ Population on October 5, 2023.

^b^ Calculated from the proportion of patients with a positive HDV RNA test result.

Inputs for the Markov model include parameters depicting the natural history of HDV infection. The proportion of patients with F4/CC was based on the proportion of patients with non‐decompensated cirrhosis from a recent real‐world study publication (Table 2) [6]. The relative distribution of patients within individual F0–F3 states was based on a publication by Romeo et al. [25]. As earlier patient identification is expected with increased HDV screening, the distribution of patients from F0 to F3 was assumed to increase by 10% with double reflex testing. Progression, treatment response, and treatment discontinuation rates were based on literature reviews, real‐world data, and expert consensus (Table 2) [23]. An annual rate of HBsAg seroclearance was assumed at 1.13% for patients off‐treatment and for those receiving HDV treatment (Table 2) [23]. Responder rates for bulevirtide at 24 and 48 weeks were based on published results from MYR 203 and MYR 301 (Table 3) [24, 39]. The responder rates for PEG‐IFN‐α at 24 (0%) and 48 weeks (14%) were based on unpublished internal data [29]. Of note, the responder rate of 14% at 48 weeks was based on an internally conducted systematic literature review and network meta‐analysis which evaluated the relative effectiveness on achieving the combined response endpoint in patients treated with bulevirtide versus PEG‐IFN‐α. As long‐term clinical data for bulevirtide in HDV are not available, patients remaining on treatment were assumed to have lifelong decreases in HDV RNA and normalisation of ALT, in line with the lower rates of advanced liver disease observed among real‐world patients who received bulevirtide, as well as the association between HDV RNA‐negativity and reduced progression to advanced liver disease [3, 40, 41, 42, 43]. Of note, model validation regarding clinical data was outside the scope of this analysis.

**TABLE 2:** Annual HDV natural history health state transitions (Markov model).

Health state		Probability		References
Baseline patient distribution		Current practice	Double reflex testing	
Non‐cirrhosis	F0	17.78%	19.56%	Current practice: Gish et al. [6]; F0–F3 reweighted per Romeo et al. [25] Double reflex testing: assumption; 10% relative increase for F0–F3
	F1	17.78%	19.56%	
	F2	19.12%	21.03%	
	F3	27.12%	29.83%	
F4		18.19%	10.01%	
**From** ^a^	**To**			
Fx	Fx+1	15.07%		Papathepdoris et al. [30]; HR = 3.0 per Da et al. [31]
F0‐F2	HCC	1.38%		Chen et al. [32]; HR = 2.77 per Alfaiate et al. [33]
F3	HCC	2.86%		Dienstag et al. [34]
CC (F4)	DCC	10.67%		Dakin et al. [35]; HR = 2.2 per Fattovich et al. [36]
	HCC	6.24%		Dakin et al. [35]; HR = 2.77 per Alfaiate et al. [33]
	LD	7.26%		Fattovich [37]; HR = 2.0 per Fattovich et al. [36]
DCC	HCC	7.83%		Dakin et al. [35]; HR = 2.77 per Alfaiate et al. [33]
	LT	1.55%		Dakin et al. [35]
	LD	15.60%		Fattovich [37]
HCC	LT	1.55%		Dakin et al. [35]
	LD	56.00%		
LT	LD	21.00%		
PLT	LD	5.70%		
**sAg seroclearance**				
Spontaneous Clearance		1.13%		Zhou [38]

Abbreviations: CC, compensated cirrhosis; DCC, decompensated cirrhosis; Fx, fibrosis stage x; LD, liver‐related death; LT, liver transplant; PLT, post‐liver transplant; sAg, surface antigen.

^a^ Uncontrolled infection.

**TABLE 3:** HDV response distributions by treatment (Markov model).

Response	Bulevirtide	PEG‐IFN‐α	References
24‐week efficacy (composite endpoint)			
Responder	34.70%	0%	Bulevirtide: weighted average of Wedemeyer et al. [24, 39] PEG‐IFN‐α: 0% responder rate per data on file [29]
Suboptimal responder	20.40%	0%	
Non‐responder	44.90%	100%	
48‐week efficacy (composite endpoint)			
Responder	44.90%	14.00%	Bulevirtide: weighted average of Wedemeyer et al. [24, 39] PEG‐IFN‐α: data on file [29]
Suboptimal responder	28.60%	0%	
Non‐responder	26.50%	86.00%	

Abbreviation: PEG‐IFN‐α, pegylated interferon‐alpha.

### 2.3 Outcomes Evaluated

The base case analysis evaluated the total number of patients diagnosed with HDV with current practice compared with double reflex testing, and the number of patients subsequently treated. Under base case assumptions, HDV‐RNA positive patients received PEG‐IFN‐α treatment in current practice and bulevirtide in double reflex testing. Additional outcomes were rates of liver‐related complications (CC, DCC, HCC, LT) and liver‐related mortality over 5 years, under each screening regimen.

Scenario analyses compared outcomes when treatment, treatment rates, or HBV prevalence were varied. Additional scenarios varied the HDV antibody screening rate and the proportion of patients receiving treatment, that is, a scenario where baseline values were assumed for both, scenarios where HDV antibody screening was increased (to 100% or 53.27% [5× from base case]) without increasing treatment rates, a scenario where treatment rates were increased but not HDV antibody screening, and a scenario where both screening and treatment rates were increased. Altogether, 10 scenarios were considered: (i) patients were treated with PEG‐IFN‐α in both screening regimens; (ii) a lower proportion (65%) of patients diagnosed under double reflex testing were treated with bulevirtide; (iii) a higher proportion (10%) of patients diagnosed under current practice were treated with PEG‐IFN‐α; (iv) increased prevalence of 0.45% of HBsAg‐positive individuals, based on published US epidemiology data [44], (v) 100% (vs. 10.65%) of patients were exposed to HDV antibody screening under current practice; (vi) 53.27% (vs. 10.65%) of patients were exposed to HDV antibody screening under current practice; (vii) 100% (vs. 25%) of patients were exposed to HDV RNA screening under current practice; (viii) 100% of patients were exposed to both HDV antibody (vs. 10.65%) and RNA screening (vs. 25%) under current practice; (ix) 85% patients were treated with PEG‐IFN‐α (vs. bulevirtide) under double reflex testing; and (x) 1.14% patients were treated with bulevirtide (vs. PEG‐IFN‐α) under current practice. A one‐way sensitivity analysis (OWSA) was included that focused on screening parameters in the analysis as aspects of disease progression on model results have been evaluated as part of other studies; throughout the OWSA, parameters were varied by ±10%.

## 3 Results

### 3.1 Number of HDV Diagnoses

**FIGURE 2:**
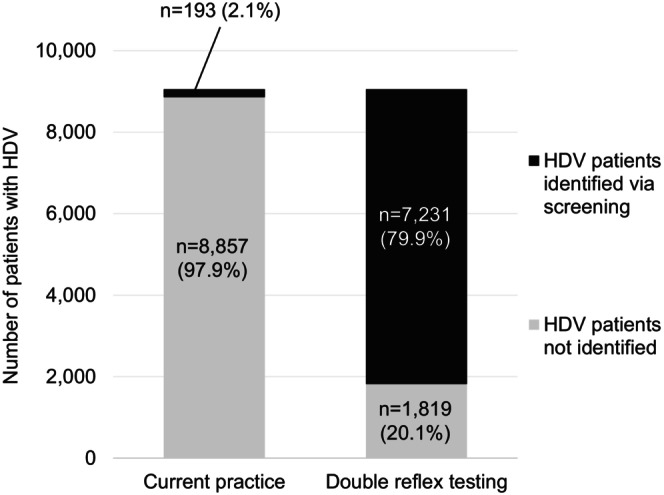
Total number of HDV diagnoses identified from current practice versus double reflex testing. HDV, hepatitis D virus.

Under current practice, an estimated 8857 patients with HDV would not be diagnosed with HDV in the United States while approximately 193 patients would be diagnosed (Figure 2). Among the 193 HDV patients diagnosed under current practice, 2 patients were treated. With the implementation of double reflex testing, 1819 patients would not be diagnosed with HDV while an estimated 7231 patients would be diagnosed (+3655% of patients diagnosed versus current practice, Figure 2). Among the 7231 HDV patients diagnosed under double reflex testing, 6146 patients were treated.

The number of patients diagnosed or undiagnosed with HDV under scenario analyses is shown in Table S1; total HDV patient numbers were only increased in the scenario where HBV prevalence was increased to 0.45%.

### 3.2 Liver‐Related Outcomes

In the base case, the number of occurrences of all liver‐related outcomes over a 5‐year time horizon is lower with double reflex testing than with current practice (Table 4). The total number of liver‐related events was 3708 events with double reflex testing and 4862 events under current practice (−24% difference). The difference in number of CC events with double reflex testing was −7%, DCC −40%, HCC −23%, LT −34%, and liver‐related deaths −32%, compared with current practice.

**TABLE 4:** Number of liver‐related outcomes occurring in current practice versus double reflex testing, base case analysis.

Distribution of events, *n*	CC (F4)	DCC	HCC	LT	LD	Total events
Current practice (+PEG‐IFN‐α^a^)						
Total	1506	769	1083	36	1468	4862
HDV patients identified via screening	32	16	23	1	31	103
HDV patients not identified	1474	753	1060	35	1437	4758
Double reflex screening (+bulevirtide^a^)						
Total	1404	458	829	24	992	3708
HDV patients identified via screening	1071	344	630	18	751	2814
HDV patients not identified	333	115	199	6	241	894

Abbreviations: CC, compensated cirrhosis; DCC, decompensated cirrhosis; LD, liver‐related death; LT, liver transplant; PEG‐IFN‐α, pegylated interferon‐alpha.

^a^ Treatment received under each screening regimen.

Results of scenario analyses are shown in Table S2. When patients under both screening regimens were treated with PEG‐IFN‐α, the number of occurrences of most liver‐related outcomes was lower for double reflex testing than for current practice. The total number of liver‐related events was 4259 events with double reflex testing and 4862 events under current practice (−12% difference). The difference in the number of CC events with double reflex testing was +6%, DCC −30%, HCC −12%, LT −24%, and liver‐related deaths −22%, compared with current practice. When the treatment rate for bulevirtide under double reflex testing was lowered from 85% to 65%, the difference in the number of CC events with double reflex testing was −3%, DCC −37%, HCC −20%, LT −31%, and liver‐related deaths −29%, compared with current practice (−20% difference in total number of liver‐related events). Conversely, when the treatment rate for PEG‐IFN‐α under current practice was increased from 1.14% to 10%, the difference in the number of CC events with double reflex testing was −7%, DCC −40%, HCC −23%, LT −34%, and liver‐related deaths −32%, compared with current practice (−24% difference in total number of liver‐related events). In the scenario where HBV prevalence increased from 0.25% to 0.45% for both current practice and double reflex testing, the difference in the number of CC events with double reflex testing was −7% (*n* = 182 less patients with double reflex testing), DCC −40% (*n* = 555), HCC −23% (*n* = 452), LT −34% (*n* = 22), and liver‐related deaths −32% (*n* = 850), compared with current practice (−24% difference in total number of liver‐related events; *n* = 1154). Increasing the proportion of patients under current practice who were exposed to HDV antibody (to 100% or 53.27%) and/or HDV RNA screening (to 100%) had minimal impact on the number of total liver‐related outcomes (−0.06% to 0% difference in total number of liver‐related events vs. base case), with results of one‐way sensitivity analyses for the scenario where 100% of patients received HDV antibody and RNA screening shown in Figure S1. There was also little difference when patients were treated with bulevirtide instead of PEG‐IFN‐α under current practice (−0.23% difference vs. base case). When patients under double reflex testing received PEG‐IFN‐α instead of bulevirtide, the total number of liver‐related events increased to 4258 (+15% difference from base case).

## 4 Discussion

HDV infection is an underdiagnosed condition that leads to more aggressive liver disease progression compared to infection with HBV alone [5, 6, 8, 9]. A previous study modelled the natural history of HDV infection and showed that bulevirtide treatment resulted in improved clinical outcomes versus best supportive care, and greater benefit was seen when treatment was started early [23]. The current study is the first to model the potential impact of implementing double reflex testing for HDV in the United States versus current practice and compared, using a two‐step Screen and Treat model, the number of HDV diagnoses and clinical outcomes under each screening approach.

Improving HDV detection rates can be achieved by reflexively screening patients with HBV for HDV antibodies and then performing quantitative HDV RNA testing in all patients who are anti‐HDV positive [5]. The current model predicted that implementing such a double reflex testing approach among all HBsAg‐positive patients would increase the number of diagnosed HDV patients by 3655% in the United States, from 193 cases under current practice to 7231 cases under double reflex testing. These results are consistent with other studies conducted in Spain and Italy, which showed that implementing reflex HDV antibody testing led to considerable increases in the HDV screening rate and number of diagnosed HDV patients [18, 19]. Rates of HDV screening in the US are presently low, with cohort studies of patients with chronic HBV reporting HDV screening rates of 6.7%–19.7% [11, 13]. Another retrospective study of HDV screening in a United States tertiary care centre showed that up to 18% of HDV‐positive patients would have been missed if the risk‐based screening approach recommended by the AASLD was applied [12]. Reasons for low HDV screening rates in the United States may include varying guideline screening recommendations, differences in the regional availability and uniformity of testing, low HDV awareness by healthcare professionals, and perceived lack of benefit of screening due to limited treatment options for HDV [11, 12].

Our study identified ~9000 HDV patients under base case assumptions; however, others have estimated that there are likely to be ~36,000 HDV‐RNA positive patients in the United States [45]. The lower number of HDV patients in our analysis could be attributed to fewer HBsAg‐positive patients identified (~845,000 individuals), based on an assumed HBV prevalence rate of 0.25% derived from published studies [27, 28]. Additionally, our model assumed that 1.91% of HBsAg‐positive patients are anti‐HDV positive, which may be an underestimate given that other studies reported HDV prevalence of 4.2%–4.6% [6, 7]. We have addressed this in a scenario where the HBV prevalence rate was increased to 0.45%, which resulted in ~1.5 million HBsAg‐positive patients and, consequently, ~16,000 HDV patients identified. Despite the increased HDV patient numbers, clinical outcomes remained consistent with those seen in the base case analysis.

There are currently no medications approved by the United States Food and Drug Administration (FDA) for HDV, and off‐label use of PEG‐IFN‐α is based on expert guidance in patients without decompensated liver disease [46]; however, the treatment landscape for HDV in the United States may soon change. Based on promising data from the pivotal MYR301 study [24], the first‐in‐class entry inhibitor bulevirtide was approved in Europe in 2023 for the treatment of chronic HDV in HDV‐RNA positive adults with compensated liver disease and is currently recommended by the European Association for the Study of the Liver [21, 47]. In the current study, increased rates of HDV diagnosis via double reflex testing and treatment with bulevirtide was predicted to lower the total number of liver‐related outcomes by 24% over a 5‐year period compared with current practice for screening and treatment with PEG‐IFN‐α. These findings suggest that improvements in HDV diagnosis via double reflex testing resulted in the earlier capture and treatment of HDV patients and improved clinical outcomes.

We further considered a scenario where all patients were treated with PEG‐IFN‐α, regardless of screening regimen; results were consistent with the base case, apart from an increase in the number of CC cases, which is likely attributed to the fact our model structure identified when patients progress to CC but not whether they experienced response first or not. Given that treated patients can regress from CC if they respond to treatment, treated patients have another chance of experiencing disease progression to a CC event after coming off treatment. Notably, this scenario may be considered conservative as the likelihood of patients consenting to receive PEG‐IFN‐α is expected to be low; given the loss of response over time, and the associated side effects and low efficacy with PEG‐IFN‐α treatment, the impact on slowing disease progression in our model is negligible [48, 49, 50]. By contrast, treatment rates are expected to be higher with an emerging treatment such as bulevirtide (vs. PEG‐IFN‐α) based on its safety profile [24, 39]. Interestingly, increasing the PEG‐IFN‐α treatment rate from 1.14% (current practice) to 10% had minimal impact on results, likely driven by the low diagnosis and treatment rate in the current scenario, low efficacy for PEG‐IFN‐α, and anticipated loss of effect (virological relapse) for patients receiving finite duration of PEG‐IFN‐α.

There are notable benefits in treating all HDV patients diagnosed by double reflex screening, that is, anti‐HDV patients who are positive for HDV RNA. The association between increased rates of liver‐related complications and deaths among those with HDV/HBV co‐infection compared with people with HBV monoinfection is well documented [1, 2, 3, 4, 5]. In particular, a recent meta‐analysis of 12 studies including a total of 4876 patients showed that HDV RNA‐positivity was associated with significantly higher risks of CC, DCC, LT, and mortality compared with patients who were HDV RNA‐negative [3]. Studies have also shown that HDV treatment resulted in lower proportions of patients who developed HCC [51], as well as preservation or improvement of liver function [40]. Recent publications reporting the proportions of patients who are able to maintain sustained HDV RNA negativity on and off bulevirtide treatment (as monotherapy, combination therapy, or long‐term treatment) further highlight the potential additional benefits beyond those currently modelled [41, 52, 53, 54, 55]. Additionally, treating patients with HDV would likely reduce the risks of HDV transmission, HBV‐related extrahepatic disease, and other HBV‐related cancers (e.g., bile duct cancer), as well as reduce stigma/discrimination and improve quality of life, as reported in patients with HCV [15, 56].

Results of this study should be considered in the context of several limitations. United States epidemiologic data on HDV are limited; although there is uncertainty regarding accurate numbers of HDV patients and of their baseline demographics and liver fibrosis stage, we have included a scenario assuming an increased HBV prevalence rate. Data regarding the natural history and impact of HDV treatment are limited. The proportions of patients accepting HDV antibody and RNA screening were assessed via expert opinion and may not be generalizable to all United States providers. There are currently no treatments for HDV approved by the US FDA; hence, our base case model, which assumed all patients diagnosed under double reflex testing would be treated with bulevirtide, reflects a future state where bulevirtide is commercially available in the US. Treatment rates for HDV under current practice and double reflex screening were based on real‐world data or assumptions; however, we have included scenarios to examine whether a reduced rate of bulevirtide treatment under double reflex testing or an increased rate of PEG‐IFN‐α treatment under current practice would impact clinical outcomes. In a future state where approved therapies are available which have higher efficacy than bulevirtide, the benefit of the double reflex testing approach would be expected to be even greater than the modelled results in the current study. Assessing the feasibility of a broad reflex‐testing approach for HDV in the United States was also beyond the scope of this study. Additionally, operational changes are likely needed to facilitate the large increase in rates of patients receiving treatment (from 1.14% in current practice to 85% in double reflex testing), which are not accounted for in this study.

This simulation study showed that implementation of double reflex HDV testing among people with HBV resulted in the earlier detection of HDV patients; modelled outcomes predicted an increase in numbers of patients diagnosed and treated, and a reduction in rates of progression to advanced liver complications and deaths. These findings are consistent with a recent cost‐effectiveness analysis which showed that one‐time universal testing and subsequent treatment for HDV among patients with chronic HBV in the United States led to a potential reduction in cirrhosis and HCC cases, and HDV‐related deaths compared with a current screening rate of 12.9% [57]. Increased detection of HDV, together with the availability of long‐term efficacy and safety data on novel and emerging treatments (e.g., bulevirtide), would likely improve HDV management, reduce rates of disease progression, and contribute towards easing the clinical and economic burden of disease in the United States. The current Screen and Treat model for HDV was informed by best available data; however, future studies are warranted to further elucidate the impact of double reflex testing.

## Conflicts of Interest

R.J.W. reports research grants to his institution from Gilead Sciences Inc., Exact Sciences, Durect Corporation, Theratechnologies, and Madrigal Pharmaceuticals, and has served as a consultant to Gilead Sciences Inc, Salix Pharmaceuticals, and Mallinkrodt Pharmaceuticals, all without compensation. R.G.G. reports grants from Gilead Sciences Inc.; serves as a consultant and advisor for Abbott, EIT pharmaInc., Fujifilm Wako Diagnostics, Genlantis, Gerson Lehrman Group, Gilead Sciences Inc., Helios, HepaTx, HepQuant, Intercept, Quest, Topography Health, and Venatorx; serves on scientific or clinical advisory boards for Genlantis, Gilead Sciences Inc., Helios, HepaTx, HepQuant, Intercept, and Prodigy; serves as the chair of the clinical advisory board for Prodigy; partner in clinical trials alliance with Topography Health; serves on the data safety monitoring boards for is part of the speakers bureau for Gilead Sciences Inc., and Intercept; is a minor stock shareholder in Riboscience and Cocrystal; and has stock options for Angiocrine, Eiger, Genlantis, HepaTx, and HepQuant. I.M.J. reports research funding to his institution from AbbVie, Assembly Biosciences, Bristol Myers Squibb, Eli Lilly, Enanta, Gilead Sciences Inc., Intercept, Janssen, Merck, and Novo Nordisk; and receiving consulting fees from Aligos, Arbutus, Arrowhead, Assembly Biosciences, Galmed, Gilead Sciences Inc., GSK, Intercept, Janssen, Merck, Roche, Takeda, and VBI Vaccines. J.K.L. has served as a consultant for Gilead Sciences Inc. M.R. and C.K. are employees of Gilead Sciences Inc. and may own stock in Gilead Sciences Inc. N.S., C.K.‐M. and H.M. have served as consultants for Gilead Sciences Inc.

## Supporting information


**Data S1:** jvh70119‐sup‐0001‐DataS1.docx.

## Data Availability

The authors have nothing to report.
