# Supplementary material for: Impact of Double Reflex Testing and Linkage to Treatment on Clinical Outcomes of Chronic Hepatitis Delta Virus Infection in the United States
Source: J Viral Hepat. 2025 Dec 17;33(1):e70119. doi: 10.1111/jvh.70119 (PMC12710454; doi:10.1111/jvh.70119)
Supplement: Supplementary file 1 — Data S1: jvh70119‐sup‐0001‐DataS1.docx. [file JVH-33-0-s001.docx]

# Impact of Double Reflex Testing and Linkage to Treatment on Clinical Outcomes of Chronic Hepatitis Delta Virus Infection in the United States

Robert J. Wong, MD, Robert G. Gish, MD, Ira M. Jacobson, MD, Joseph K. Lim, MD, Marvin Rock, DrPH, Csilla Kinyik-Merena, MS, Hanxiao Ma, MS, Nathaniel Smith, PhD, Chong Kim, PhD

# Supplementary Results

Supplementary Table 1. Total number of HDV diagnoses identified from current practice vs double reflex testing, base case and scenario analyses

| **Base case / Scenario**^†^ | **Screening regimen** | **HDV patients diagnosed via screening, n (%)** | | **HDV patients not identified, n (%)** | **Total, N** |
| --- | --- | --- | --- | --- | --- |
|  |  | **HDV patients identified** | **HDV patients treated** |  |  |
| **Base case** | Current practice | 193 (2.1%) | 2 (1.1%) | 8,857 (97.9%) | 9,050 |
|  | Double reflex testing | 7,231 (79.9%) | 6,146 (85.0%) | 1,819 (20.1%) | 9,050 |
| **Scenarios** | | | | | |
| **All patients treated with PEG-IFN-α** | Current practice | 193 (2.1%) | 2 (1.1%) | 8,857 (97.9%) | 9,050 |
|  | Double reflex testing | 7,231 (79.9%) | 6,146 (85.0%) | 1,819 (20.1%) | 9,050 |
| **65% patients treated with bulevirtide under double reflex testing** | Current practice | 193 (2.1%) | 2 (1.1%) | 8,857 (97.9%) | 9,050 |
|  | Double reflex testing | 7,231 (79.9%) | 4,700 (65.0%) | 1,819 (20.1%) | 9,050 |
| **10% patients treated with PEG-IFN-α under current practice** | Current practice | 193 (2.1%) | 19 (10.0%) | 8,857 (97.9%) | 9,050 |
|  | Double reflex testing | 7,231 (79.9%) | 6,146 (85.0%) | 1,819 (20.1%) | 9,050 |
| **0.45% HBsAg-positive patients** | Current practice | 344 (2.1%) | 4 (1.1%) | 15,817 (97.9%) | 16,161 |
|  | Double reflex testing | 12,912 (79.9%) | 10,976 (85.0%) | 3,248 (20.1%) | 16,161 |

^†^ Unless otherwise stated, patients under current practice received treatment with PEG-IFN-α and patients under double reflex testing received treatment with bulevirtide.
HBsAg, hepatitis B surface antigen; PEG-IFN-α, pegylated interferon-alpha.

Supplementary Table 2. Number of liver-related outcomes occurring in current practice versus double reflex testing, scenario analyses

| **Scenario** | **Distribution of events, n** | **CC (F4)** | **DCC** | **HCC** | **LT** | **LD** | **Total events** |
| --- | --- | --- | --- | --- | --- | --- | --- |
| **All patients treated with PEG-IFN-α** | **Current practice (+PEG-IFN-α**^†^**)** | | | | | | |
|  | **Total (as per base case)** | **1,506** | **769** | **1,083** | **36** | **1,468** | **4,862** |
|  | HDV patients identified via screening | 32 | 16 | 23 | 1 | 31 | 103 |
|  | HDV patients not identified | 1,474 | 753 | 1,060 | 35 | 1,437 | 4,758 |
|  | **Double reflex screening (+PEG-IFN-α**^†^**)** | | | | | | |
|  | **Total** | **1,593** | **541** | **951** | **27** | **1,147** | **4,259** |
|  | HDV patients identified via screening | 1,260 | 427 | 751 | 21 | 906 | 3,366 |
|  | HDV patients not identified | 333 | 115 | 199 | 6 | 241 | 894 |
| **65% patients treated with bulevirtide under double reflex testing** | **Current practice (+PEG-IFN-α^a^)** | | | | | | |
|  | **Total (as per base case)** | **1,506** | **769** | **1,083** | **36** | **1,468** | **4,862** |
|  | HDV patients identified via screening | 32 | 16 | 23 | 1 | 31 | 103 |
|  | HDV patients not identified | 1,474 | 753 | 1,060 | 35 | 1,437 | 4,758 |
|  | **Double reflex screening (+bulevirtide**^†^**)** | | | | | | |
|  | **Total** | **1,464** | **485** | **868** | **25** | **1,041** | **3,882** |
|  | HDV patients identified via screening | 1,131 | 370 | 668 | 19 | 800 | 2,988 |
|  | HDV patients not identified | 333 | 115 | 199 | 6 | 241 | 894 |
| **10% patients treated with PEG-IFN-α under current practice** | **Current practice (+PEG-IFN-α**^†^**)** | | | | | | |
|  | **Total** | **1,506** | **769** | **1,083** | **36** | **1,468** | **4,861** |
|  | HDV patients identified via screening | 32 | 16 | 23 | 1 | 31 | 103 |
|  | HDV patients not identified | 1,474 | 753 | 1,060 | 35 | 1,437 | 4,758 |
|  | **Double reflex screening (+bulevirtide**^†^**)** | | | | | | |
|  | **Total (as per base case)** | **1,404** | **458** | **829** | **24** | **992** | **3,708** |
|  | HDV patients identified via screening | 1,071 | 344 | 630 | 18 | 751 | 2,814 |
|  | HDV patients not identified | 333 | 115 | 199 | 6 | 241 | 894 |
| **0.45% HBsAg-positive patients** | **Current practice (+PEG-IFN-α**^†^**)** | | | | | | |
|  | **Total** | **2,689** | **1,373** | **1,933** | **64** | **2,622** | **8,682** |
|  | HDV patients identified via screening | 57 | 29 | 41 | 1 | 56 | 185 |
|  | HDV patients not identified | 2,632 | 1,344 | 1,892 | 63 | 2,566 | 8,497 |
|  | **Double reflex screening (+bulevirtide**^†^**)** | | | | | | |
|  | **Total** | **2,508** | **818** | **1,481** | **42** | **1,772** | **6,621** |
|  | HDV patients identified via screening | 1,913 | 614 | 1,125 | 32 | 1,342 | 5,025 |
|  | HDV patients not identified | 595 | 205 | 356 | 10 | 431 | 1,596 |
| **100% patients exposed to Ab screening under current practice** | **Current practice (+PEG-IFN-α**^†^**)** | | | | | | |
|  | **Total** | **1,506** | **769** | **1,083** | **36** | **1,468** | **4,861** |
|  | HDV patients identified via screening | 301 | 153 | 216 | 7 | 293 | 970 |
|  | HDV patients not identified | 1,205 | 615 | 866 | 29 | 1,175 | 3,891 |
|  | **Double reflex screening (+bulevirtide**^†^**)** | | | | | | |
|  | **Total (as per base case)** | **1,404** | **458** | **829** | **24** | **992** | **3,708** |
|  | HDV patients identified via screening | 1,071 | 344 | 630 | 18 | 751 | 2,814 |
|  | HDV patients not identified | 333 | 115 | 199 | 6 | 241 | 894 |
| **53.27% patients exposed to Ab screening under current practice** | **Current practice (+PEG-IFN-α**^†^**)** | | | | | | |
|  | **Total** | **1,506** | **769** | **1,082** | **36** | **1,468** | **4,860** |
|  | HDV patients identified via screening | 160 | 82 | 115 | 4 | 156 | 516 |
|  | HDV patients not identified | 1,346 | 687 | 967 | 32 | 1,312 | 4,345 |
|  | **Double reflex screening (+bulevirtide**^†^**)** | | | | | | |
|  | **Total (as per base case)** | **1,404** | **458** | **829** | **24** | **992** | **3,708** |
|  | HDV patients identified via screening | 1,071 | 344 | 630 | 18 | 751 | 2,814 |
|  | HDV patients not identified | 333 | 115 | 199 | 6 | 241 | 894 |
| **100% patients exposed to RNA screening under current practice** | **Current practice (+PEG-IFN-α**^†^**)** | | | | | | |
|  | **Total** | **1,506** | **769** | **1,083** | **36** | **1,468** | **4,862** |
|  | HDV patients identified via screening | 128 | 65 | 92 | 3 | 125 | 414 |
|  | HDV patients not identified | 1,378 | 703 | 991 | 33 | 1,343 | 4,448 |
|  | **Double reflex screening (+bulevirtide**^†^**)** | | | | | | |
|  | **Total (as per base case)** | **1,404** | **458** | **829** | **24** | **992** | **3,708** |
|  | HDV patients identified via screening | 1,071 | 344 | 630 | 18 | 751 | 2,814 |
|  | HDV patients not identified | 333 | 115 | 199 | 6 | 241 | 894 |
| **100% patients exposed to both Ab screening and RNA screening under current practice** | **Current practice (+PEG-IFN-α**^†^**)** | | | | | | |
|  | **Total** | **1,505** | **768** | **1,082** | **36** | **1,467** | **4,859** |
|  | HDV patients identified via screening | 1,203 | 614 | 865 | 29 | 1,172 | 3,882 |
|  | HDV patients not identified | 303 | 155 | 218 | 7 | 295 | 977 |
|  | **Double reflex screening (+bulevirtide**^†^**)** | | | | | | |
|  | **Total (as per base case)** | **1,404** | **458** | **829** | **24** | **992** | **3,708** |
|  | HDV patients identified via screening | 1,071 | 344 | 630 | 18 | 751 | 2,814 |
|  | HDV patients not identified | 333 | 115 | 199 | 6 | 241 | 894 |
| **85% patients treated with PEG-IFN-α under double reflex testing** | **Current practice (+PEG-IFN-α**^†^**)** | | | | | | |
|  | **Total (as per base case)** | **1,506** | **769** | **1,083** | **36** | **1,468** | **4,862** |
|  | HDV patients identified via screening | 32 | 16 | 23 | 1 | 31 | 103 |
|  | HDV patients not identified | 1,474 | 753 | 1,060 | 35 | 1,437 | 4,758 |
|  | **Double reflex screening (+PEG-IFN-α**^†^**)** | | | | | | |
|  | **Total** | **1,593** | **541** | **950** | **27** | **1,147** | **4,258** |
|  | HDV patients identified via screening | 1,260 | 426 | 751 | 21 | 906 | 3,365 |
|  | HDV patients not identified | 333 | 115 | 199 | 6 | 241 | 894 |
| **1.14% patients treated with bulevirtide under current practice** | **Current practice (+bulevirtide**^†^**)** | | | | | | |
|  | **Total** | **1,503** | **767** | **1,080** | **36** | **1,465** | **4,851** |
|  | HDV patients identified via screening | 1,200 | 613 | 863 | 29 | 1,170 | 3,874 |
|  | HDV patients not identified | 303 | 155 | 218 | 7 | 295 | 977 |
|  | **Double reflex screening (+bulevirtide**^†^**)** | | | | | | |
|  | **Total (as per base case)** | **1,404** | **458** | **829** | **24** | **992** | **3,708** |
|  | HDV patients identified via screening | 1,071 | 344 | 630 | 18 | 751 | 2,814 |
|  | HDV patients not identified | 333 | 115 | 199 | 6 | 241 | 894 |

Varied parameter(s) is shown as underlined. ^†^ Treatment received under each screening regimen and scenario.
CC, compensated cirrhosis; DCC, decompensated cirrhosis; LD, liver-related death; LT, liver transplant; PEG-IFN-α, pegylated interferon-alpha.

Supplementary Figure 1. OWSA for scenario where proportion exposed to HDV antibody screening and proportion exposed to HDV RNA screening increased to 100%


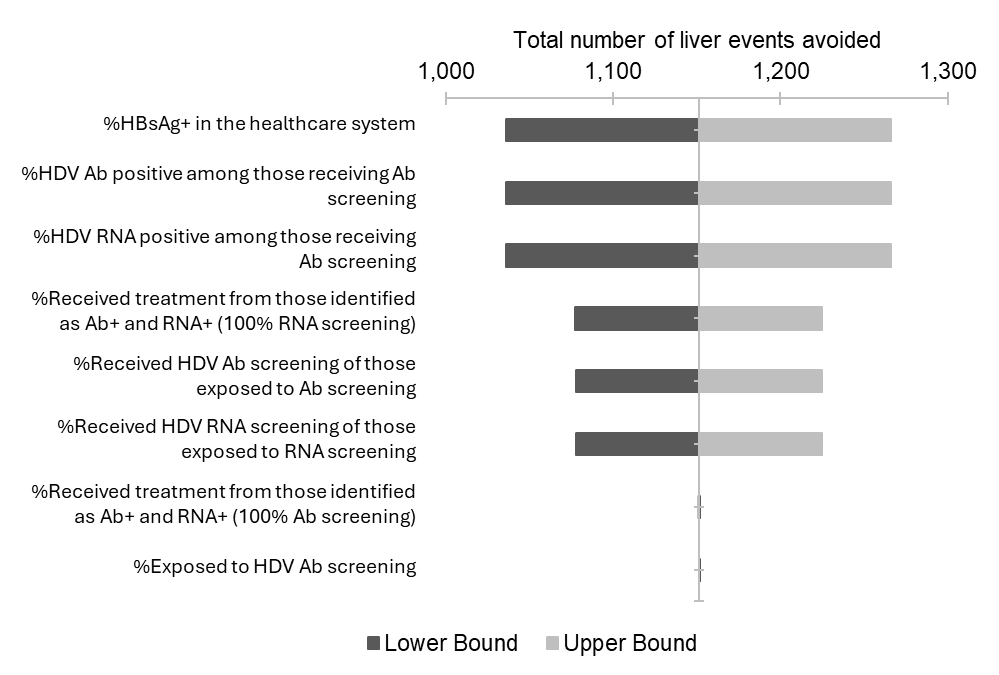


Screening inputs were varied by ±10% of the base value.
Ab, antibody; OWSA, one-way sensitivity analysis
